# Oral health Status of special Olympics athletes in the UAE: findings from annual dental screenings

**DOI:** 10.3389/froh.2026.1811492

**Published:** 2026-04-24

**Authors:** Asma H. AlNababteh, Salma B. AlBahnasawi, Areen Abu Hijleh, Anas Salami

**Affiliations:** 1 School of Health Sciences, Hamdan Bin Mohammed Smart University, Dubai, United Arab Emirates; 2 Clinical Services Department, Appolonia World Dentistry, Abu Dhabi, United Arab Emirates; 3 Health Programs Section, UAE Special Olympics Foundation, Abu Dhabi, United Arab Emirates; 4 Hamdan Bin Mohammed College of Dental Medicine, Dubai Health, Mohammed Bin Rashid University of Medicine and Health Sciences, Dubai, United Arab Emirates

**Keywords:** athletes, intellectual disability, oral health, special Olympics, United Arab Emirates

## Abstract

**Objective:**

To assess the oral health status and treatment needs of Special Olympics athletes with intellectual disabilities (ID) in the United Arab Emirates (UAE), addressing a data gap in the Gulf Cooperation Council (GCC) region.

**Methods:**

A cross-sectional study was conducted in accordance with STROBE guidelines, using data from 817 Special Olympics athletes across 10 events in the UAE between September 2022 and July 2025. After excluding 161 incomplete records, 656 forms were analyzed. Data were collected using the standardized Special Olympics Healthy Athletes System (HAS) form. Trained and calibrated dentists performed visual oral examinations to assess oral hygiene habits, signs of gingival disease, presence of untreated decay, oral pain, and treatment urgency. Descriptive statistics with 95% confidence intervals and chi-square tests were used to assess gender-based stratification.

**Results:**

The cohort of 656 athletes had a median age of 20 years, with 68% being male. Over one-third (36%) reported not brushing daily. Clinical examination revealed high rates of oral disease: 67% presented with signs of gingivitis (95% CI: 63.4%–70.6%) and 64% had untreated dental decay (95% CI: 60.3%–67.7%). Furthermore, 20% reported oral pain (95% CI: 17.0%–23.2%). A substantial majority (72.1%) required dental care.

**Conclusion:**

Special Olympics athletes in the UAE exhibit a significant burden of untreated dental decay and gingivitis, reflecting substantial unmet treatment needs. These findings, the first comprehensive data from this population in the UAE, highlight a critical need for targeted prevention programs and enhanced access to specialized dental care.

## 1 Introduction

Dental screening is an essential component of preventive oral healthcare, supporting early disease detection, timely intervention, and education on effective hygiene practices. Evidence from recent systematic reviews demonstrates that school-based dental screening programs can identify untreated dental disease, promote referrals for care, and improve oral-health outcomes when combined with appropriate follow-up services (1). Routine dental checkups help prevent dental caries, periodontal disease, and other oral conditions that can affect nutrition, speech, and overall quality of life. For individuals with intellectual disabilities—who frequently encounter anatomical, cognitive, and behavioral challenges—regular dental screening is particularly important, as they are disproportionately affected by poor oral health and unmet treatment needs (2).

The World Health Organization emphasizes that standardized survey methods for oral-health data are crucial for planning and evaluating effective programs (3). In its Global Oral Health Action Plan, WHO further highlights screening and early detection as key strategies, noting that early identification reduces treatment burden and cost while strengthening health systems by identifying high-risk groups and tailoring preventive services (4). Despite these global health promotion initiatives, oral diseases remain highly prevalent among individuals with ID worldwide, underscoring the gap between policy aspirations and clinical reality.

Intellectual disability (ID) affects millions of people worldwide, presenting with varying levels of cognitive and adaptive impairment (5). Under the Convention on the Rights of Persons with Disabilities, individuals with ID are entitled to equitable healthcare standards and protection from discrimination (6). Nevertheless, despite global improvements in medicine and public health, people with ID continue to experience poorer overall health outcomes, including significantly worse oral health (2). They are especially susceptible to oral diseases due to biological and behavioral factors. Motor impairments, craniofacial differences, reduced manual dexterity, and difficulties performing daily hygiene often result in elevated plaque levels, gingivitis, dental caries, and periodontal disease (7). Behavioral challenges and reliance on caregivers may hinder consistent oral care, while communication difficulties can delay the reporting of pain, contributing to the progression of untreated disease (8). Emerging evidence suggests that sensory-adaptive dental environments can reduce anxiety and improve cooperation (8), yet such approaches remain insufficiently implemented.

To address these challenges, the Special Olympics established the Special Smiles program as part of its Healthy Athletes initiative. The program provides free dental screenings, individualized oral hygiene education, preventive treatments, and referrals for follow-up care (9, 10). Special Smiles also collects standardized oral health data from athletes with ID worldwide, creating one of the most extensive global datasets for this population. Previous screening events have consistently revealed high rates of gingivitis, dental trauma, and untreated caries (9, 10).

At the Special Olympics World Games in Berlin (2023), trained and calibrated dentists conducted comprehensive oral examinations, behavioral assessments, and evaluations of access to care among athletes with intellectual disabilities from numerous countries (10). Findings from the event showed that many athletes had untreated caries, gingival inflammation, and limited access to preventive services, underscoring persistent oral health inequities (10). The Berlin dataset also enables regional comparisons, revealing ongoing gaps in care for underrepresented areas, such as the Gulf Cooperation Council (GCC).

Although the Special Smiles program has a global presence, there remains a notable data gap in the GCC region, where few studies have investigated the oral health of individuals with ID. This lack of region-specific evidence makes it challenging to assess needs accurately and develop effective oral health policies.

Therefore, the aim of this study is to assess the oral health status and treatment needs of Special Olympics athletes with ID who have participated in Special Smiles screenings in the United Arab Emirates (UAE). Specifically, the objectives of this study were to:

1. determine the prevalence of untreated dental decay, signs of gingival disease, and self-reported oral pain among Special Olympics athletes in the UAE;
2. assess the self-reported daily oral hygiene practices of these athletes;
3. evaluate the level and urgency of dental treatment needs; and
4. explore whether oral health outcomes differ by gender.

## 2 Materials and methods

This study is reported in accordance with the Strengthening the Reporting of Observational Studies in Epidemiology (STROBE) guidelines for cross-sectional studies (11).

### 2.1 Study design and setting

A cross-sectional study was conducted utilizing data from 10 Special Olympics UAE “Special Smiles” screening events held between September 2022 and July 2025. The screenings were part of the broader Special Olympics Healthy Athletes® program. The screening process involved athlete registration, a standardized oral examination, oral hygiene instruction, and the application of topical fluoride varnish.

### 2.2 Participants and recruitment

Participants were Special Olympics athletes with a confirmed diagnosis of an intellectual disability, as determined by assessments from a senior psychiatrist and psychologist. All athletes resided with their families; none lived in institutional settings. Athletes were recruited from designated sports clubs for individuals with special needs across the UAE and were invited to participate in the screening events by the Special Olympics UAE Health Programs Department.

### 2.3 Data sources and measurement

Oral health data were collected using the standardized Special Olympics Healthy Athletes System (HAS) Special Smiles data collection form, which is used globally across all Special Smiles programs to ensure data consistency. Each screening event involved at least six trained dentists, with two supervising clinical directors authorized by Special Olympics International present at all times to ensure protocol adherence. Prior to each event, all examiners underwent a calibration session based on the Training Manual for Standardized Oral Health Screening, developed by Special Olympics in collaboration with the U.S. Centers for Disease Control and Prevention (CDC), to ensure consistent application of diagnostic criteria. Due to the logistical constraints of mass-screening events involving athletes with ID, formal inter-examiner reliability testing was not conducted; however, the standardized training protocol and continuous oversight by clinical directors helped mitigate examiner variability.

Examinations were conducted using disposable dental mirrors, flashlights, and 2 × 2 gauze. Personal protective equipment was worn by all screeners, and only disposable instruments were used. Key variables included demographic information (age, gender), self-reported daily brushing habits, and clinical findings. “Untreated decay” was defined as the visual presence of at least one area of cavitation accommodating a 0.5 mm diameter or larger on any primary or permanent tooth. “Signs of gingival disease” were recorded if redness, swelling, or abnormal contour of the gingiva was observed in three or more locations. Self-reported oral pain and treatment urgency were also recorded. Other data points recorded on the HAS form, including malocclusion, fluorosis, dental injury, and missing teeth, were omitted from this analysis to maintain focus on the primary outcomes and to avoid potential reporting bias inherent in mass screening events.

### 2.4 Statistical analysis

The collected data were entered into MS Excel, cleaned for inconsistencies, and exported to IBM SPSS Statistics (Version 31) for analysis. Age was calculated based on the reported year of birth and the date of examination. As the primary aim of this study was to provide a foundational, descriptive assessment of oral health status in a previously un-researched population, the analysis was focused on descriptive statistics. Frequencies, percentages, and median were used to summarize demographic characteristics and key oral health outcomes. Ninety-five percent confidence intervals (95% CIs) were calculated for key prevalence estimates using the Wilson score method. Chi-square tests were used to assess for significant differences in outcomes stratified by gender. Age was categorized into five groups (0–10, 11–20, 21–30, 31–40, and ≥41 years) to align with categories used in Special Olympics global health data reporting, thereby facilitating international comparisons. Records with any missing key variable were excluded from the analysis.

### 2.5 Ethical consideration

This study involves human participants and was conducted in accordance with the Declaration of Helsinki. Written informed consent was obtained from all participating athletes or their legal guardians prior to the screening. Ethical approval was obtained from the Institutional Review Board of Mohammed Bin Rashid University (Number: IRB-2025-922).

## 3 Results

**Figure 1:**
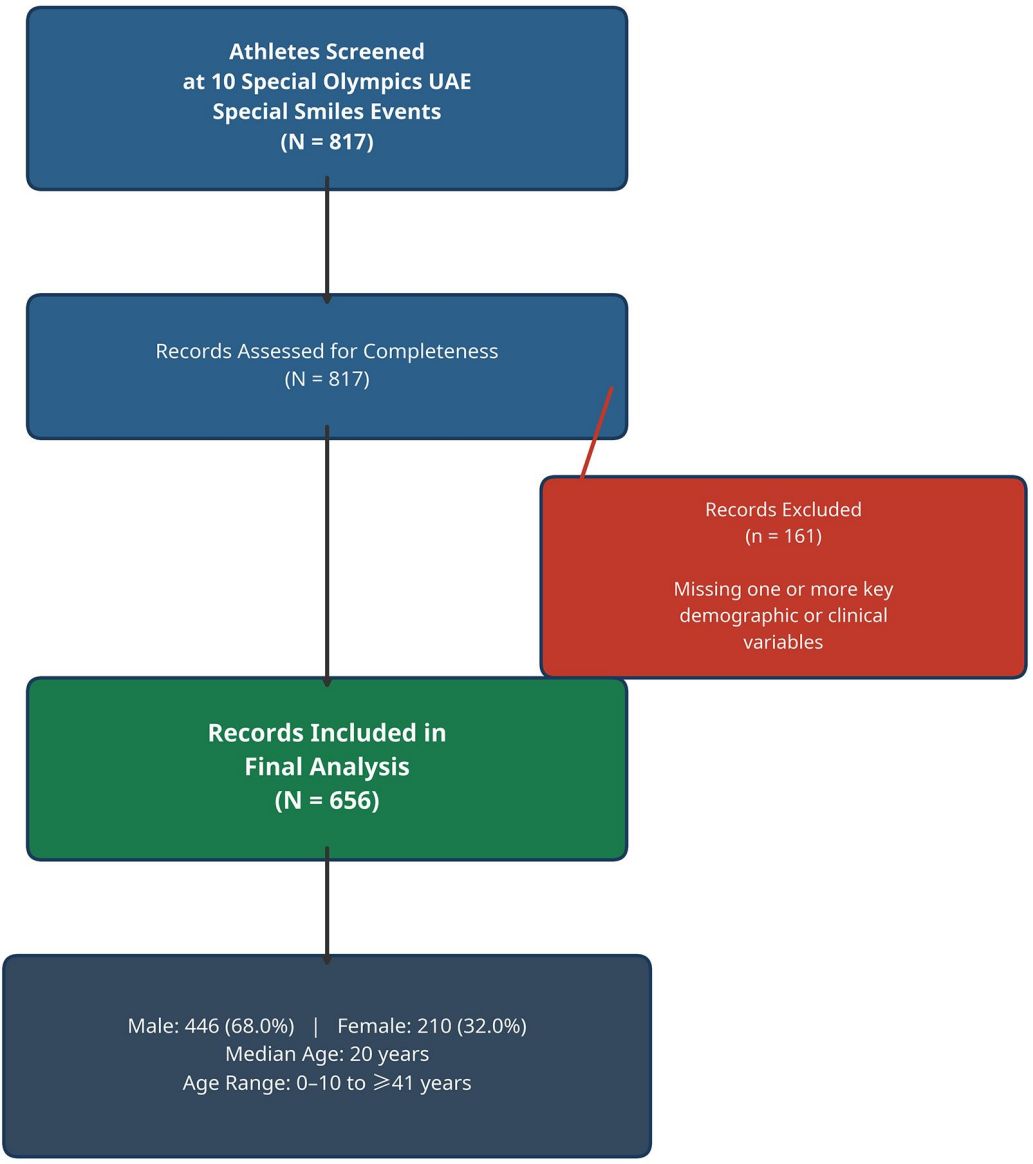
Participant flow diagram.

A total of 817 athletes were screened across 10 events, with 656 individuals having complete records and subsequently included in the final analysis. There were 161 records excluded, representing 19.7% of the total, due to the absence of essential demographic or clinical outcome variables. A flow diagram illustrating participant inclusion is provided in Figure 1. The final cohort exhibited a median age of 20 years, with the majority of participants being male at 68%. The predominant age group was 11–20 years, comprising 46.0% of the participants. Detailed demographic and oral health characteristics are available in Table 1.

**Table 1:** Demographic and oral health Status of special Olympics athletes in the UAE (*N* = 656).

Variables	Athletes screened *n* = 656 (Valid %)
Sociodemographic	
*Gender*	
Female	210 (32.0%)
Male	446 (68.0%)
*Age group*	
Median (years)	20
<10 years	59 (9.0%)
11–20 years	302 (46.0%)
21–30 years	182 (27.7%)
31–40 years	86 (13.1%)
>41 years	27 (4.1%)
Screening results	
*Oral hygiene status*	
Daily brushing (Yes)	422 (64.3%)
Daily brushing (No)	234 (35.7%)
*Oral health outcomes*	
Pain in mouth (Yes)	132 (20.1%)
Signs of gingival disease (Yes)	439 (67.0%)
Untreated decay (Yes)	421 (64.1%)
*Dental treatment needs*	
Urgent dental care	58 (8.8%)
Non-urgent dental care	415 (63.3%)
Maintenance visits	183 (27.9%)

Clinical examination revealed a high prevalence of oral disease. A majority of athletes (67%; 95% CI: 63.4%–70.6%) presented with signs of gingival disease, and a similarly high proportion (64.1%; 95% CI: 60.3%–67.7%) had untreated dental decay. Furthermore, 20.1% (95% CI: 17.0%–23.2%) of athletes reported mouth pain at the time of screening. Only 64.3% (95% CI: 60.6%–68.0%) reported brushing their teeth daily.

**Figure 2:**
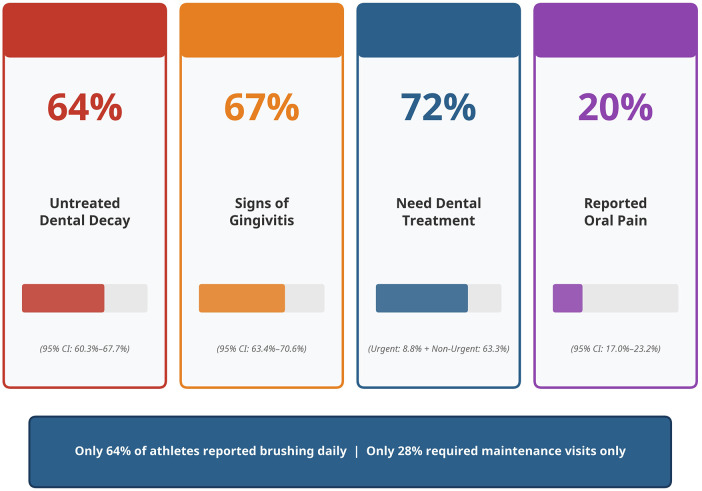
Key oral health findings. Special Olympic athletes in the UAE (*N* = 656).

Assessment of treatment urgency indicated a substantial need for dental care. A combined 72.1% of athletes required either non-urgent (63.3%; 95% CI: 59.6%–67.0%) or urgent (8.8%; 95% CI: 6.6%–11.0%) dental care. Only 27.9% (95% CI: 24.5%–31.3%) were deemed to require routine maintenance visits only. A summary of the key oral health findings is presented in Figure 2.

Stratification by gender (Table 2) revealed no statistically significant differences in the prevalence of untreated decay (*p* = 0.58), gingival disease signs (*p* = 0.43), or oral pain (*p* = 0.57) between male and female athletes.

**Table 2:** Oral health outcomes stratified by gender.

Outcome	Female (*n* = 210)	Male (*n* = 446)	*p*-value
Untreated decay	138 (65.7%)	283 (63.5%)	0.58
Signs of gingival disease	145 (69.0%)	294 (65.9%)	0.43
Pain in the mouth	45 (21.4%)	87 (19.5%)	0.57
Daily brushing (Yes)	130 (61.9%)	292 (65.5%)	0.38

## 4 Discussion

This study, the first of its kind in the UAE, reveals a significant burden of unmet oral health needs among Special Olympics athletes with ID. The findings confirm that a majority suffer from preventable oral diseases, with 67% showing signs of gingivitis and 64% having untreated decay. These results are alarming, directly address the regional data gap highlighted in the introduction, and provide a crucial baseline for public health action.

The prevalence of untreated caries (64%) in the UAE cohort is a stark indicator of health inequity. This figure is consistent with a meta-analysis of children with IDD in India (64%) (12) and aligns with findings from the broader MENA region, which has been identified as having a high burden of untreated decay (9). While direct cross-study comparisons must be made with caution due to potential differences in screening protocols, populations, and healthcare systems, the rate in our cohort is substantially higher than the 22% reported in Belgium (13) but similar to the 60% found in Pakistan (14). The high rate of decay in the UAE, despite its advanced healthcare system, suggests that significant barriers may prevent this population from receiving timely and effective treatment. This interpretation is consistent with the conclusion from the Berlin Games data, which found that even annual dental visits did not significantly reduce the prevalence of untreated caries among athletes with ID, underscoring the need for more specialized and effective care models (10).

Similarly, the high prevalence of gingivitis (67%) points to considerable challenges with daily oral hygiene. This rate is comparable to the 70.4% reported among Romanian athletes and higher than the rates reported in France (49.5%), Italy (62.2%), and Turkey (66.3%) (15). These findings are consistent with global data showing higher rates of gingival disease in the MENA region (9). This clinical finding is likely compounded by the low rate of daily brushing (64%), a figure lower than the over 80% reported in most other regions (9). As noted in the introduction, factors such as reduced manual dexterity, reliance on caregivers, and behavioral challenges directly impact the ability to perform daily hygiene, making this a key area for intervention (8, 9).

Poor oral health has a tangible impact on well-being that extends beyond the oral cavity. Untreated dental disease can lead to chronic pain, difficulty eating, and poor nutrition, which in turn can negatively affect an athlete's general health, quality of life, and ability to train and compete effectively (16, 17). The finding that 20% of athletes reported being in pain at the time of screening is a particularly distressing indicator of advanced disease and delayed care. This rate is higher than the 12.7% reported at the 2023 World Games in Berlin (10) and reinforces the point that communication difficulties in this population can hinder timely symptom reporting (8).

A combined 72.1% of athletes required either non-urgent or urgent dental care, aligning with global data showing consistently high unmet treatment needs (10). These data suggest that existing care models may be inadequate for this population and point to the need for more accessible, specialized, and sensory-adapted dental services (8).

This study's primary significance lies in its contribution of crucial, region-specific data. As mandated by the WHO and the Convention on the Rights of Persons with Disabilities, equitable access to healthcare is a fundamental right (4, 6). The data presented here provide the evidence needed to advocate for and design programs that fulfill this right for athletes with ID in the UAE.

Looking forward, future research should build on this descriptive baseline. Longitudinal studies are needed to establish causal relationships between risk factors and oral health outcomes. The inclusion of a neurotypical control group would allow for a precise quantification of disparities. Analytical studies employing logistic regression could identify specific determinants of poor oral health. Furthermore, qualitative research is essential to explore the specific barriers to care from the perspectives of athletes, families, and caregivers to inform the development of more effective and culturally appropriate interventions.

### 4.1 Limitations

Several limitations should be considered when interpreting the findings of this study. First, the cross-sectional design allows for prevalence assessment but does not permit causal inference between risk factors and oral health outcomes. Second, the convenience sample of Special Olympics athletes may not be fully representative of all individuals with ID in the UAE, as athletes who participate in such events may have different functional abilities or more supportive social networks compared to the broader ID population (11). Third, visual examination without radiographs may have led to an underestimation of the true prevalence of dental caries, particularly interproximal or incipient lesions. Fourth, the data on oral hygiene habits were self-reported or caregiver-reported, which introduces the possibility of recall or social desirability bias. Fifth, the exclusion of 19.7% of records due to missing data may introduce selection bias, although some degree of incomplete data is expected in large-scale volunteer-led screening events. Finally, the lack of formal inter-examiner reliability assessment is a limitation, though it was mitigated by the standardized training protocol and continuous oversight by authorized clinical directors.

## 5 Conclusion

Special Olympics athletes in the UAE exhibit a high prevalence of untreated dental decay and gingivitis, reflecting a significant burden of preventable oral disease and substantial unmet treatment needs. These findings, representing the first comprehensive data from this population in the GCC region, fill a critical data gap and provide a strong evidence base for action. There is an urgent need to develop targeted oral health prevention programs, enhance access to specialized and inclusive dental care, and provide tailored education for athletes, their families, and healthcare professionals.

## Data Availability

The raw data supporting the conclusions of this article will be made available by the authors, without undue reservation.
